# Are Static Spacers Superior to Articulated Spacers in the Staged Treatment of Infected Primary Knee Arthroplasty? A Systematic Review and Meta-Analysis

**DOI:** 10.3390/jcm11164854

**Published:** 2022-08-18

**Authors:** Michele Fiore, Andrea Sambri, Matteo Filippini, Lorenzo Morante, Claudio Giannini, Azzurra Paolucci, Claudia Rondinella, Renato Zunarelli, Pierluigi Viale, Massimiliano De Paolis

**Affiliations:** 1 Orthopaedics and Traumatology Unit, IRCCS Azienda Ospedaliera, Universitaria di Bologna, 40138 Bologna, Italy; 2 Infectious Disease Unit, IRCCS Azienda Ospedaliera, Universitaria di Bologna, 40138 Bologna, Italy

**Keywords:** knee arthroplasty, periprosthetic joint infection, two-stage protocol, static spacer, articulated spacer, knee revision surgery

## Abstract

In the treatment of knee periprosthetic joint infection with a two-stage protocol, static spacers allow for the local delivery of high doses of antibiotics and help to preserve soft tissue tension. Articulated spacers were introduced to better preserve flexion after the reimplantation. The aim of this systematic review is to provide a comprehensive data collection of the results of these different spacers. An in-depth search on the main clinical databases was performed concerning the studies reporting data on the topic. A total of 87 studies and 4250 spacers were included. No significant differences were found both in pooling data analysis and meta-analysis of comparative studies about infection recurrences, complications, and clinical scores. Mean active knee flexion at last follow-up after total knee reimplantation was found to be significantly higher using articulated spacers (91.6° ± 7° for static spacers vs. 100.3° ± 9.9° for articulated spacers; *p* < 0.001). Meta-analysis also recognized this strong significant difference (*p* < 0.001). This review has confirmed that articulated spacers do not appear to be inferior to static spacers regarding all clinical outcomes, while they are superior in terms of active flexion. However, the low quality of the studies and the risk for selection bias with complex patients preferentially treated with static spacers need to be accounted for.

## 1. Introduction

Periprosthetic joint infection (PJI) is one of the main complications following primary total knee arthroplasty (TKA), with high morbidity and a significant negative impact on the outcome. Because of the increase in the number of arthroplasties, the incidence of PJI has been increasing steadily as well, and it has been reported to range from 0.5% to 1.9%, currently representing a growing social and economic issue for health systems [1,2]. Treatment of PJI represents one of the main challenges of modern orthopedics, requiring a multidisciplinary approach, as it aims for infection control, pain relief, and restoration of joint function [3].

The main treatment options for primary PJIs include debridement and implant retention (DAIR), even with the use of local adjuvants (Debridement, Antibiotic Pearls, and Retention of the Implant—DAPRI) [4], single-stage revision, one-and-half revision with long-lasting spacer, and two-stage revision. Currently, there are well-established guidelines for the management of infections after knee arthroplasty, with DAIR recommended only for early infections with an immature bacterial biofilm and exchange of the prosthesis required for late infection or in case of implant loosening [5,6]. In particular, two-stage treatment has proven to be the most cross-adaptive and the most recommended in cases of infections with highly virulent bacteria and/or bone or soft tissue problems (with possible fistulas) [6,7,8,9]. The two-stage procedure consists of the removal of the infected prosthesis and cement, followed by extensive debridement of the non-viable tissue and multiple washes. A temporary spacer impregnated with antibiotic is then implanted and left in place for a variable amount of time. Postoperatively, long-term antibiotic therapy is set up. Empirical intravenous broad-spectrum antibiotic therapy is generally begun immediately after surgery. After culture results, antibiotic therapy is modified, using an oral regimen whenever possible [10].

The reimplantation is performed once the infection has been eradicated, on the basis of clinical and laboratory criteria. However, if there is any suspicion of persistent infection, a repeat debridement with exchange of the spacer should be undertaken.

The use of an antibiotic-loaded cement spacer is an established method to increase knee stability and for local antibiotic administration prior to implantation of the definitive prosthesis [11,12,13,14]. Many different types of spacers are regularly used in surgical practice. Static spacers have demonstrated excellent results over the years in terms of eradicating the infection and therefore remain a valid treatment option [8]. However, several risks associated with the use of the static spacer are described in the literature, such as reduced function between the two stages, shortening of the soft tissues, increased bone loss, and an increased risk of spacer displacement [15]. To overcome these problems, articulated spacers are increasingly used, which allow one to avoid the shortening of soft tissues, to reduce bone loss, and to guarantee the patient a better function between the two stages [16]. Several categories of mobile spacers are regularly used, including prefabricated cement-on-cement components, intraoperatively molded cement-on-cement and cement-on-polyethylene components, and autoclaved femoral component on polyethylene [17].

Currently, the scientific evidence to support the use of static or articulated spacers is still not conclusive, both for the functional outcomes and the infection eradication rates. Thus, the choice is often determined by the surgeon’s experience.

The aim of this systematic literature review is to provide a comprehensive data collection on two-stage reimplantation using different types of spacers, in terms of infection control, complications, and functional outcomes.

## 2. Materials and Methods

This systematic review was conducted in accordance with the 2020 PRISMA guidelines (Preferred Reporting Items of Systematic Reviews) [18].

All studies (randomized controlled trials (RCT), prospective (PCCS) and retrospective comparative studies (RCCS), prospective (PCS) and retrospective case series (RCS)) reporting the use of static or articulated cement or hybrid metal/cement/polyethylene spacers in two-stage surgery to treat PJI of a primary knee arthroplasty were included. The two-stage surgical protocol consists of the following: (1) a first surgery with total removal of the infected implant, extended surgical debridement, and placement of a spacer (usually an antibiotic-laden spacer)—this step may be repeated in the case of failure to control the infection; (2) a second surgery for further debridement and reimplantation (regardless of the type of implant used).

No restrictions were made based on the initial indication for knee replacement surgery, as the study focused on the treatment outcome of a complication, namely the PJI. Due to the wide time window of the studies included in this review, the definition of PJI has not been uniformly stated. The criteria adopted by the individual authors for the most recent studies are those which were discussed in the 2018 International Consensus Meeting on Orthopedic Infections [5]. With regard to the remaining articles, the authors of this review unanimously agreed that the criteria adopted by the authors of the individual studies included in this review were always diagnostically appropriate to identify patients with plausible PJI. Otherwise, non-conforming studies were excluded.

Studies reporting the results of PJI treatments other than two-stage protocols (including DAIR with or without partial component replacement, single-stage with partial or total explantation, one-and-half procedure with long-lasting spacer, permanent spacer, megaprosthesis, and resection arthroplasty) were excluded. Studies reporting the results of various treatments of knee PJI were excluded. Cases in which single- or two-stage protocols were used in the treatment of a recurrence of infection were excluded. Only studies with a minimum follow-up of 12 months and a minimum of 5 patients were included. Biomechanical studies, cadaveric studies, “in vitro” studies, and animal model studies were excluded. Only studies in English were included.

**Figure 1:**
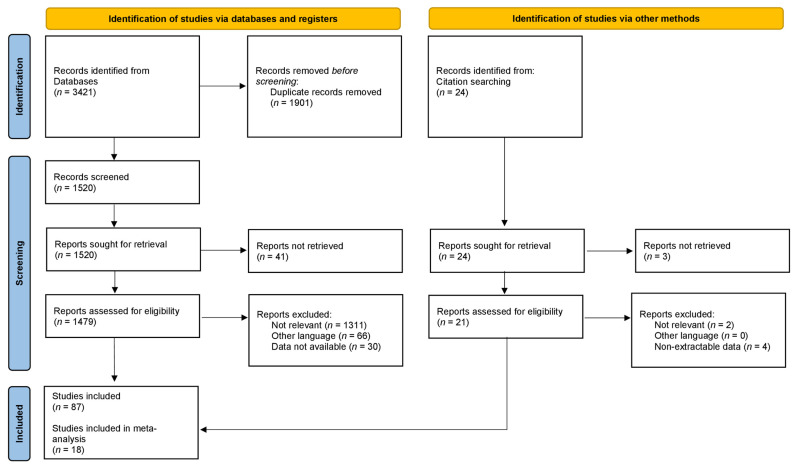
PRISMA 2020 flow diagram and the selection of studies.

**Figure 2:**
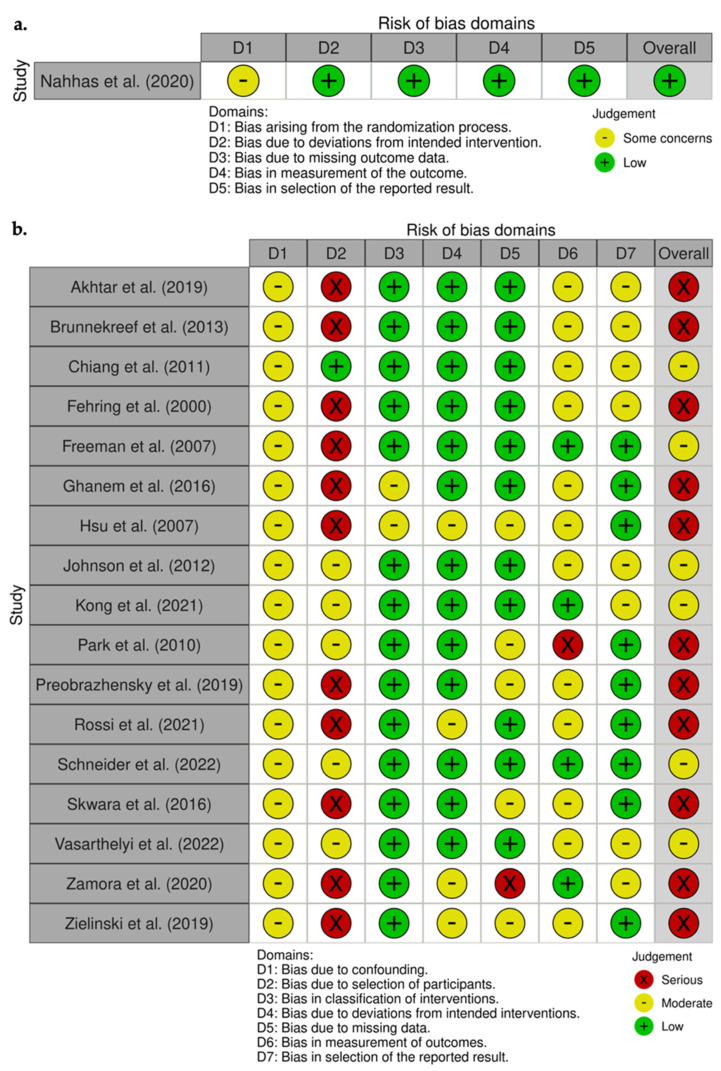

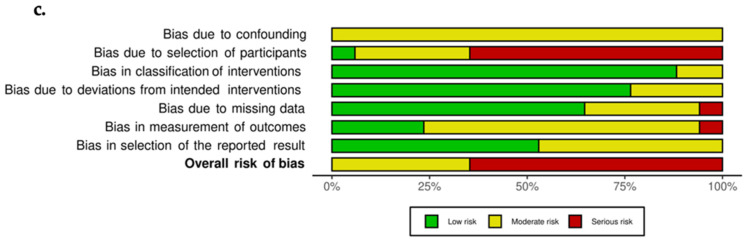
Quality assessment of the included studies in meta-analysis according to RoB2 [43] (**a**) and ROBINS-I [21,23,25,29,30,31,34,38,39,44,46,47,48,50,52,54,106] (**b**) tools: “traffic light” plots of the domain-level judgments for each individual result (**a**,**b**); weighted bar plots of the distribution of risk-of-bias judgments within each bias domain (**c**).

Studies eligible for this systematic review were identified through an electronic systematic search of the studies published from 1 January 2000 up to 30 June 2022, published on PubMed (https://pubmed.ncbi.nlm.nih.gov/ (accessed on 30 June 2022)), Scopus (https://www.scopus.com (accessed on 30 June 2022)), and Web of Science (www.webofscience.com (accessed on 30 June 2022)) databases. Terms used for the search included “infection”, “prosthesis-related infection”, “knee joint”, “knee arthroplasty”, “knee replacement”, “periprosthetic infection”, “2-stage”, “two-stage”, “explant”, “re-implantation”, “static spacer”, “articulated spacer”, “mobile spacer”, “dynamic spacer”. Articles that were considered relevant by electronic search were retrieved in full text, and a cross-referencing search of their bibliographies was performed, to find further related articles. Reviews and meta-analyses were also analyzed, in order to broaden the search to studies that might have been missed through the electronic search. All duplicates were removed, and all the articles retrieved have been analyzed. After the first screening, records without eligibility criteria were excluded (Figure 1). Remnant studies were categorized by type, according to the Oxford Centre for Evidence-Based Medicine (OCEBM). To assess the quality of the articles, the revised Cochrane risk-of-bias tool for randomized trials (RoB2) (Figure 2a) and the Cochrane risk of bias in non-randomized studies of interventions (ROBINS-I) assessment tool (Figure 2b) were utilized [19,20]. Each study was assessed by two reviewers (Ma.F. and L.M.) independently and in duplicate; disagreement was resolved by the senior author (M.D.). All the included studies were analyzed, and data related to topics of interest were extracted and summarized (Table 1 and Table 2).

In detail, data extracted included study type, mean age, mean follow-up, number and details of spacers, mean time to infection onset, bacterial populations, number of spacers used, and repeated first stages, mean time between first stage and second stage, mean duration of antibiotic therapy, number of PJI recurrences, number of cases in which no reimplantation was performed, mean active knee flexion at last follow-up, functional outcome at last follow-up, and peri-operative non-infection-related local complications. Functional outcomes were reported according to the most reported scoring systems used in the studies analyzed in this review: Knee Society Score (KSS) and Hospital for Special Surgery Knee-Rating Scale (HSS). Local peri-operative complications not related to infection were reported, including extensor lag, spacer subluxation/fracture, extensor mechanism rupture, nerve palsy, periprosthetic fracture, dislocation, instability, arthrofibrosis, hematoma, and delayed wound healing. Success of the treatment was defined as the achievement of infection control at last follow-up (the absence of clinical and/or radiological and/or laboratory signs of infection, as mentioned in the individual papers). Failure of the treatment was defined as the persistence of infection, re-infection, or no reimplantation; the repetition of the first stage of the two-stage protocol due to persistence of infection was not considered a failure when it eventually resulted in successful control of the infection at last follow-up after the end of the treatment.

**Figure 3:**
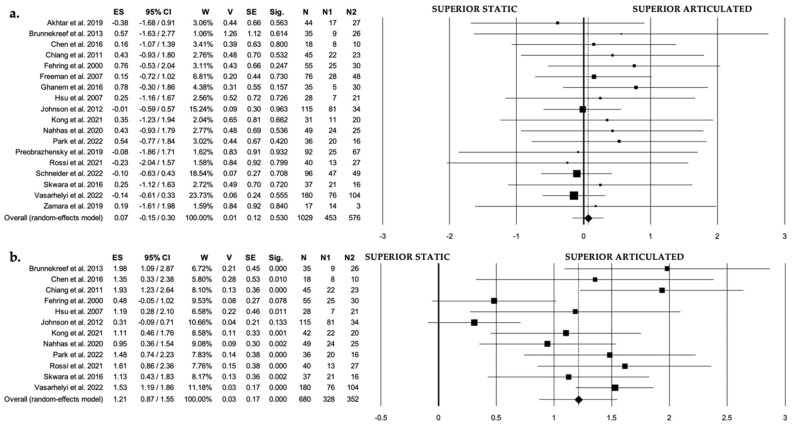

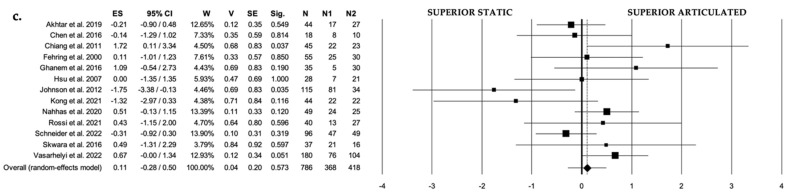
Forest plot of overall meta-analysis evaluating comparative studies (static spacers vs. articulated spacers) with data about PJI recurrences [21,23,24,25,29,30,31,34,38,39,43,44,46,47,48,50,52,54] (**a**), active knee flexion at last follow-up (**b**), and non-infection-related peri-operative local complications (**c**). Abbreviations: ES, effect size; 95% CI, 95% confidence interval; W, weight; V, variance; SE, standard error; N, sample size; N1, static spacer series sample size; N2, articulated spacer series sample size.

**Figure 4:**
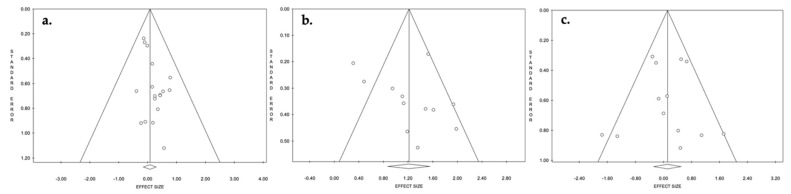
Funnel plot of effect sizes for publication bias of the comparative studies evaluating PJI recurrences (**a**), active knee flexion at last follow-up (**b**), and non-infection-related peri-operative local complications (**c**).

Studies with reported quantitative data were used for statistical analysis (Table 3 and Table 4). Weighted means and standard deviations were calculated to summarize the values reported in the individual studies and to compare them. Chi-square statistics (Pearson Chi-square, Yates Chi-Square, Fisher exact test, Fisher–Freeman–Halton test) were used to assess associations and homogeneity among categorical variables. For quantitative variables, the Shapiro–Wilk test was used to verify normal distribution. The Levene test was used to assess the equality of variances. As a parametric test, the two-tailed unpaired Student T-test was used in case of equality of the variances; otherwise, the Welch T-test was used. The Mann–Whitney U-test was used as a non-parametric test in case of non-normal distribution of the variables. Spearman’s rho was used to identify monotonic correlations between variables. Only comparative studies were included in the meta-analysis (Figure 3 and Figure 4). Quantification of the extent of statistical heterogeneity across studies included in the meta-analysis employed the inconsistency statistic (I^2^ > 75% was considered as high heterogeneity). Potential sources of heterogeneity by study level and clinically relevant characteristics were explored using stratified analysis and meta-regression. Publication bias was assessed using Egger’s regression symmetry test. *p*-value < 0.05 was considered to be significant. All statistical analyses were performed with IBM SPSS v26.0 for MacOS (SPSS Inc., Chicago, Illinois) and ProMeta 3 (Internovi, Cesena, Italy) software.

## 3. Results

A total of 3421 studies were found through the electronic search and 21 studies were added after the cross-referenced research on the bibliographies of the examined full-text articles. After a preliminary analysis, a total of 87 studies reporting series of knee spacers used for two-stage treatment of PJI were included in this systematic review (1 randomized controlled trial, 3 prospective comparative cohort studies, 26 retrospective comparative cohort studies, 9 prospective case series, 48 retrospective case series [18,19,20,21,22,23,24,25,26,27,28,29,30,31,32,33,34,35,36,37,38,39,40,41,42,43,44,45,46,47,48,49,50,51,52,53,54,55,56,57,58,59,60,61,62,63,64,65,66,67,68,69,70,71,72,73,74,75,76,77,78,79,80,81,82,83,84,85,86,87,88,89,90,91,92,93,94,95,96,97,98,99,100,101,102] (Table 1 and Table 2; Figure 1)). Among these, eighteen studies were included in the meta-analysis (Figure 3 and Figure 4). Studies comparing static vs. articulated spacers were 1 RCT [43], 1 PCCS [22], and 16 RCCS [18,20,21,26,27,28,31,35,36,41,43,44,45,47,49,51]. In total, 34 series on static spacers [18,19,20,21,22,23,24,25,26,27,28,29,30,31,32,33,34,35,36,37,38,39,40,41,42,43,44,45,46,47,48,49,50,51] and 71 on articulated spacers [18,20,21,26,27,28,31,35,36,41,43,44,45,47,49,51,52,53,54,55,56,57,58,59,60,61,62,63,64,65,66,67,68,69,70,71,72,73,74,75,76,77,78,79,80,81,82,83,84,85,86,87,88,89,90,91,92,93,94,95,96,97,98,99,100,101,102] were found (Figure 3).

The overall quality of the studies included in the meta-analysis, assessed by the RoB2 and the ROBINS-I tools [16,17], was high in only one case [40], moderate in 6 cases [22,27,35,36,45,49], and low in 11 cases [18,20,21,26,28,31,41,43,44,47,51] (Figure 2a–c).

A total of 4250 knee spacers were included: 1511 static spacers and 2739 articulated spacers (Table 3). The two groups were highly homogeneous considering a number of variables (Table 3). Mean age was comparable between static and articulated series (67 ± 5.6 years and 66.4 ± 3.5, respectively; *p* = 0.532) (Table 3). No statistical difference was found between the two groups also concerning the mean follow-up (68 ± 52.3 months for static group and 53.5 ± 32.9 months for articulated group; *p* = 0.117) (Table 3). The most frequent bacterial populations found were *Staphylococcus aureus*, *Streptococcus epidermidis*, and other coagulase-negative staphylococci in both groups (Table 3). Mean time to PJI onset from primary arthroplasty surgery was also similar (34.8 ± 14.3 months for static group and 36.8 ± 11.9 months for articulated group; *p* = 0.735) (Table 3). No significant difference was found in the time between first and second stage (3.1 ± 1.1 months for static group and 3.6 ± 2.3 months for articulated group; *p* = 0.480), nor in the number of spacer exchanges with repeated first stage before reimplantation (5.4% vs. 4% for static and articulated series, respectively; *p* = 0.159) (Table 3). Instead, a mismatch was found between the two groups in the mean duration of post-operative antibiotic therapy after first-stage surgery, being longer for static spacers (7.2 ± 1.9 vs. 6.1 ± 1 weeks; *p* = 0.007) (Table 3).

With respect to the results in terms of infection control, a significantly higher rate of both no reimplantation and PJI recurrence was found when static spacers were used. In detail, a revision knee arthroplasty was not performed in 7.1% of the PJIs in the static spacer group and in 4.3% of the cases in the articulated group (*p* = 0.001), while PJI recurrence was found in 12.4% vs. 9% of the two-stage procedures (*p* = 0.001) (Table 3). The time elapsed between the first and second stage appeared to directly influence the PJI recurrence rate (with a trend towards more recurrences in the case of longer time with a spacer in situ), although a significant correlation was only found for articulated spacers (*p* = 0.040) (Table 4). The meta-analysis performed on comparative studies evaluating the PJI recurrence with static vs. articulated spacers confirmed a trend for better infection control using articulated spacers, although no significant difference was found between the groups (*p* = 0.530) (Figure 3a). No significative heterogeneity (I^2^ ≈ 0%, *p* = 0.992) or relevant publication bias (Figure 4a) was found regarding the PJI recurrence rate. No significant difference was found concerning the mean time to PJI recurrence between static and articulated spacers (13.7 ± 3.9 months and 23.2 ± 12.1 months, respectively; *p* = 0.125) (Table 3). Furthermore, no correlations between the mean time to second stage after spacer placement and the mean time to PJI recurrence were found (Table 4).

With regard to the functional outcomes, mean active knee flexion at last follow-up was found to be significantly higher using articulated spacers (91.6° ± 7° for static spacers vs. 100.3° ± 9.9° for articulated spacers; *p* < 0.001) (Table 3). The meta-analysis also confirmed this strong significant difference (*p* < 0.001) (Figure 3b). Moderate heterogeneity (I^2^ = 69.1%, *p* = 0.059) and no relevant publication bias (Figure 4b) were found regarding the active knee flexion. A significant negative correlation was found between the mean time to second stage after spacer placement and the mean final active knee flexion, which appeared to be particularly marked when using articulated spacers (*p* = 0.019) (Table 4). However, when clinical scores were considered (KSS and HSS), no significant difference was found between static and articulated groups (81.1 ± 13.1 vs. 81.9 ± 5.5 for KSS, *p* = 0.792; 81.8 ± 0.7 vs. 81.7 ± 7 for HSS, *p* = 0.981) (Table 3).

No difference was found regarding the incidence of peri-operative local complications not related to the PJI between static and articulated spacers (complication rate: 16.7% vs. 16.5%; *p* = 0.852) (Table 3). The revision rate for non-infection-related complications was found to be also similar between static and articulated spacers (2.9% vs. 3.1%; *p* = 0.819) (Table 3). The meta-analysis did not find significant differences between the groups either (*p* = 0.573) (Figure 3c). Low heterogeneity (I^2^ = 41.3%, *p* = 0.099) and no relevant publication bias (Figure 4c) were found regarding the complication rate.

## 4. Discussion

Both static and articulated antibiotic-laden spacers have benefits and drawbacks, and the choice is based on multiple factors, including the clinical assessment of the patient’s general functional status, general health, soft tissue envelope of the knee, virulence of the organism, and extent of bone loss [107]. The existing literature on the subject largely consists of small series with evidence levels III and IV and a limited number of randomized prospective trials.

In this review, we found, in the pooled analysis, a significantly lower number of PJI recurrences when an articulated spacer was used. This trend was also found in the meta-analysis of the comparative studies alone, though without a statistically significant difference. No significant differences were found either in the number of non-infection-related complications or in the functional results from the evaluation of the HSS and KSS scores, as already reported by previous studies [11].

Conversely, a strong difference emerged in favor of articulated spacers, both in the general pooled data analysis and the meta-analysis of comparative studies, regarding active knee flexion capability at the last follow-up after prosthesis reimplantation.

The main benefit of articulated spacers is that they enable movement of the joint between surgeries. Articulated spacers also allow a more comfortable position of the knee during sitting, standing, and car travel. Maintaining motion facilitates the recovery of limb function during treatment of infection. Knee flexion preserves the length and elasticity of the extensor mechanism and helps to prevent scarring of the soft tissue around the joint and capsular stiffening [96,108,109]. As a result, the extent of surgical exposure required and the overall difficulty of the second-stage surgery can be decreased [26,34,109]. Moreover, the findings of an in vitro study showed that cyclical loading of the cement spacers enhanced the elution of vancomycin and tobramycin [110]. A broad assortment of articulated spacers that can be placed after the removal of an infected total knee arthroplasty is available—for example, (1) handmade cement-on-cement spacers without molds, (2) premolded or preformed antibiotic cement spacers (with or without stems), (3) surgical molds for intraoperative fabrication (with or without metal femoral runners), and (4) autoclaved or new metal femoral and polyethylene components (Table 1) [111]. Most of the articulated spacers included in this study were found to be cement-on-cement spacers. Consequently, no further investigation was performed to reveal whether there are differences in outcomes depending on the subtype of mobile spacer.

Common indications for use of a static spacer are (1) patients with severe uncontrolled infections; (2) ligamentous laxity, particularly in the case of collateral ligament compromise, as an articulated spacer would not allow for multiplanar knee stability; (3) extensor mechanism disruption or insufficiency, as active flexion and control of the knee would not be achieved; (4) compromised soft tissue coverage over the joint, since motion might apply additional tension; (5) severe bone loss after prosthesis explant, as they can be customized to fill the gap and eventually stabilized using intramedullary dowels [9,108,109,112,113,114]. Moreover, static spacers are usually cheaper [115,116].

However, several shortcomings of static spacers have been suggested. Several studies have reported poor limb mobility with static spacers after reimplantation compared to articulated spacers [108,117,118]. In addition, unanticipated bone loss as a result of spacer migration has been observed. Using static spacers may also complicate exposure during the second-stage procedure due to the shortening of the ligaments and quadriceps, as well as wound closure [29].

A factor that is difficult to standardize within the two-stage protocol is the time of spacer persistence, before reimplantation. Longer intervals between the two stages are known to correlate with worse infectious and functional outcomes [7,119,120]. Elution of the antibiotic from any spacer reaches its peak in the first 72 h from placement: after this time, the function of any is mainly mechanical [13,121]. Moreover, a longer time of spacer persistence may increase the incidence of mechanical complications such as spacer rupture or dislocation, which can eventually lead to an interim spacer exchange [122].

We observed that, in the case of articulated spacers, a spacer persistence of more than 3 weeks increased the number of PJI recurrences. Furthermore, it was found that spacer persistence progressively decreases the ability of articulated spacers to preserve active flexion.

It was not possible to perform a detailed analysis of any inconsistencies in terms of the surgical and infectious complexity of the cases in order to exclude any selection bias whereby the more complex cases were preferentially treated with a static spacer. For example, the study by Guild et al., analyzing data on the existence of bone loss of any type, found no statistical difference in the placement of static vs. articulating spacers for the indication of bone loss [11]. However, when they classified bone loss according to the Anderson Orthopaedic Research Institute (AORI) classification [123], they found that static spacers were placed significantly more frequently for femoral bone loss than articulated spacers [11]. These data, however, may be biased as only a small minority of the studies specifically addressed pre-existing bone loss. However, not only bone deficiency has to be considered when assessing complexity. It depends on many other factors (type of microorganism, quality of soft tissue, comorbidity, etc.), and even within individual studies, it was almost never possible to effectively differentiate cases by complexity. A possible patient selection bias among the included studies represents the major limitation of this study. A previous review by Pivec et al. attempted to divide patients with an articulated spacer into complex and non-complex cases and compared the results between these two subgroups and patients with a static spacer [124]. They reported a slightly higher PJI recurrence rate in the articulated spacer group with only complex patients compared to the static spacer group, but no statistical significance was shown [124]. In the present review, considering the wide variability of the criteria used in the individual studies and the paucity of studies in which the individual patients could be characterized, we decided not to perform such an analysis. However, this review only considered primary infections, so it is reasonable to assume that tremendously destructive conditions of the knee that are unsuitable for dynamic spacers are a minority and probably not crucial in the interpretation of the overall emerging findings, also considering the high number of spacers included in this review. Unfortunately, only high-quality studies, with accurate assessment to ensure the homogeneity of patient selection, can help to solve this issue. A review, although systematic, can only state that it is reasonable to believe that the use of articulated spacers should definitely be considered in all cases where there are no significant contraindications, as it offers excellent results with respect to infection control and functional outcomes, with complications comparable to those expected with the use of static spacers. Unfortunately, it is difficult to establish the limit beyond which the use of a static spacer can guarantee greater benefit.

Among the limitations of this article, in addition to those already mentioned, the average low quality of the studies (for the majority consisting of case series or retrospective comparative studies) must be considered. In addition, this is certainly not the first review on the topic and essentially confirms evidence that has already emerged. The main strength is the amount of data collected and the depth of the analysis. In fact, to the best of our knowledge, it is the first review to provide a large-scale quantitative analysis. These aspects make it a very comprehensive and up-to-date review on the subject and reinforce the conclusion that only high-quality studies can clarify the elements still under discussion.

## 5. Conclusions

In conclusion, this review confirms that articulated spacers do not appear to be inferior to static spacers in terms of infection control, complications, and functional results, while they are superior in terms of active flexion granted after reimplantation. Statis spacers, often mostly used in more severe cases, can offer similar infection control in this scenario. However, despite the high number of included spacers, considering the average low quality of the studies included and the impossibility of determining the presence and extent of a selection bias in the choice of the spacers, it is not possible to generalize the results that emerged. Nevertheless, in cases that meet all the appropriate conditions for the placement of articulated spacers, optimal results can be expected, and their use can be recommended.

## Figures and Tables

**Table 1:** Data from series reporting on static spacers.

Article	Level of Evidence	Study Type	Patient N° (Spacer N°)	Mean Age (Year)	Mean Follow-Up (Months)	Mean Time of PJI Onset after Implant (Months)	Most Frequent Pathogens	Spacer Exchange: Repeated First Stage	Mean Time between First Stage and Second Stage (Months)	No Reimplantation (*n*°)	PJI Recurrence (*n*°)	Mean Time to Recurrence (Months)	Mean Duration of Antibiotic Therapy (Weeks)	Active Knee Flexion at Last Follow-Up (Degrees)	Functional Outcomes at Last Follow-Up: Score Type and Values	Peri-Operative Non-Infection Related-Complications (*n*°)
Akhtar et al., 2019 [21]	3	RCCS	17	81.3	46	N/A	N/A	2	9	N/A	1	N/A	N/A	N/A	N/A	6
Barrack et al., 2000 [22]	3	PCCS	28	68.5	36 (range 24–60)	N/A	N/A	0	0.9–1.4	2	2 (artrodesi)	N/A	N/A (range 4–7)	89	KSS: 115	N/A
Brunnekreef et al., 2013 [23]	3	RCCS	9	61	12	62.4	N/A	0	3.6 (range 1–10)	0	0	/	6	73.8	N/A	N/A
Chen et al., 2016 [24]	3	RCCS	8	73.9 (range 63–82)	40.8	N/A	N/A	0	5.1 (range 1.6–13.8)	0	2	17.5	6	74.3 (range 50–90)	KSS: 71.4 (range 60–81)	2
Chiang et al., 2011 [25]	3	PCCS	22	72 (range 67–80)	N/A	N/A	N/A	1	3.1 (range 2–4)	1	2	N/A	11.7	85 (range 70–100)	HSS: 82 (range 81–88)	7
Choi et al., 2012 [26]	3	RCCS	14	N/A	N/A	N/A	N/A	0	6	4	7	N/A	6	97 (range 75–130)	N/A	11
Emerson et al., 2002 [27]	3	RCCS	26	65.7	90 (range 33.6–152.4)	N/A	N/A	0	N/A (range 6–12)	0	8	N/A	6	93.7	N/A	N/A
Faschingbauer et al., 2016 [28]	4	RCS	133	70.1 ± 9.9	N/A	N/A	N/A	0	2.8	32	16	N/A	6	N/A	N/A	20
Fehring et al., 2000 [29]	3	RCCS	25	N/A	36 (range 24–72)	N/A	N/A	0	N/A	0	3	N/A	6	98 ± 17 (range 50–120)	HSS: 83 ± 17 (range 37–98)	2
Freeman et al., 2007 [30]	3	RCCS	28	71.2	86.6 (range 24–196.3)	N/A	N/A	0	N/A	0	3	N/A	6	N/A	KSS: 45 (range 35–80)	N/A
Ghanem et al., 2016 [31]	3	RCCS	5	N/A	N/A	N/A	N/A	N/A	N/A	0	3	N/A	range 4–6	N/A	N/A	N/A
Haleem et al., 2004 [32]	4	RCS	96	69 (range 37–89)	86.4 (range 30.0–158.4)	26.2 (range 0.5–177)	26.0% MSSA; 14.6% MRSA	N/A	1.4 (range 0.2–80.4)	0	9	12 (range 1.2–117.6)	5.3 (range 1–24)	90 (range 30–120)	KSS: 89 (range 35–97)	6
Hipfl et al., 2019 [33]	4	RCS	97	70 (range 30–88)	41 (range 27–56)	N/A	42% CoNS; 22% *P. acnes*	9	2.1 (range 1.4–5.5)	0	15	10 (range 1–26)	9 (range 6–24)	N/A	N/A	10
Hsu et al., 2007 [34]	3	RCCS	7	N/A	101 (range 63–120)	N/A	N/A	0	2.7 (range 1.8–3.5)	0	1	21	9.7 (range 6–12)	78 (range 60–100)	KSS: 81.4	N/A
Hsu et al., 2008 [35]	4	RCS	32	66 (range 50–78)	68.3 (range 8–197)	N/A	15.6% *S. epidermidis*; 12.5% MSSA	0	7.4 (range 2.3–29.7)	4	4	N/A (range: 2.5–7)	range 6–8	88 ± 19 (range 30–120)	KSS: 82 ± 14 (range 33–99)	22
Husted et al., 2002 [36]	4	RCS	17	72.2 (range 60–78)	25.7 (range 5–62)	17.2 (range 1–133)	41.2% *S. aureus*; 41.2% *S. epidermidis*	0	N/A	2	2	N/A	5.4	99.3 (range 70–130)	N/A	N/A
Ippolito et al., 2021 [37]	4	RCS	21	52.4 ± 20.6	123.6 ± 76.8 (range 20.4–291.6)	57.4 (range 3–246)	24% CoNS; 19% *S. aureus*	0	N/A	1	7	N/A	12	100 ± 17	N/A	18
Johnson et al., 2012 [38]	3	RCCS	81	61 (range 58–64)	66 (range 12–121)	N/A	N/A	N/A	3.5 (range 2.7–4.3)	N/A	14	N/A	N/A	95 (range 30–130)	KSS: 84 (range 48–100)	0
Kong et al., 2021 [39]	3	RCCS	22	67.2 ± 10.1	43 (range 30–61)	N/A	N/A	1	3 (range 1.8–5.5)	0	1	N/A	N/A	80 (range 70–110)	KSS: 60 ± 6.3	0
Lichstein et al., 2016 [40]	4	RCS	109	67 (range 42–89)	44.4 (range 24.0–117.6)	N/A	51% *Staphylococcus* spp.; 19% *Streptococcus* spp.	0	N/A	N/A	7	N/A	11 (range 5–20)	100 (range 60–139)	KSS: 86 (range 65–98)	N/A
Lo Presti et al., 2021 [41]	4	RCS	12	64 (range 39–85)	34.3 (range 10–62)	N/A	25% MRSA; 16.7% *E. faecalis*	4	N/A	8	2	N/A	N/A (range 6–8)	N/A	N/A	0
Ma et al., 2020 [42]	3	RCCS	66	70.3 ± 11.0 (range 19–86)	75.3 ± 30.6 (range 24–133)	N/A	N/A	6	4	2	0	/	SHC: 0.7 STC: 14.7	N/A	N/A	5
Nahhas et al., 2020 [43]	1	RCT	24	64.9 ± 8.4	42 ± 14.4	N/A	N/A	2	2.4 ± 0.7 (range 2.1–2.6)	2	2	N/A	6	103 ± 12.7 (range 97.6–108.3)	KSS: 69.8 ± 14.1 (range 63.6–73.1)	13
Park et al., 2010 [44]	3	RCCS	20	66.5 (range 48–84)	36 (range 24–62)	N/A	30% MRSA; 20% MSSA	N/A	N/A	N/A	3	N/A	6	92 (range 65–140)	HSS: 80 (range 74–97)	N/A
Petis et al., 2019 [45]	4	RCS	240	N/A	N/A	N/A	N/A	N/A	N/A	N/A	N/A	N/A	N/A	N/A	N/A	N/A
Preobrazhensky et al., 2019 [46]	3	RCCS	25	N/A	12	N/A	N/A	0	N/A	0	0	/	N/A (range 6–8)	N/A	N/A	N/A
Rossi et al., 2021 [47]	3	RCCS	13	N/A	18	N/A	N/A	0	N/A	0	0	/	N/A	100.8 ± 28	KSS: 76.9 ± 12	1
Schneider et al., 2022 [48]	3	RCCS	47	63 (range 9–36)	N/A	N/A	N/A	N/A	N/A	2	10	N/A	N/A	90.5	N/A	6
Silvestre et al., 2013 [49]	4	RCS	43 (45)	72 (63–81)	86 (range 60–132)	N/A	17.8% *Staphylococcus* spp.; 15.6% MRSA	N/A	4.4	2	2	N/A	6	92 (range 50–115)	KSS: 83 (range 43–95)	7
Skwara et al., 2016 [50]	3	RCCS	21	N/A	8.5	N/A	N/A	N/A	N/A	N/A	2	N/A	N/A	79 ± 26	N/A	1
Springer et al., 2004 [51]	4	RCS	34	66.5 (range 48–84)	N/A	N/A	61.7% CoNS; 17.6% *S. aureus*	N/A	N/A	1	3	N/A	6	N/A	N/A	N/A
Vasarhelyi et al., 2022 [52]	3	RCCS	76	69.4 ± 10.0	228 ± 75.6	51,4 (range 3–120)	N/A	4	3	N/A	10	N/A	6	82.1 ± 25.4	KSS: 72 ± 23.3	9
Vielgut et al., 2021 [53]	4	RCS	77	64.9 (range 31.3–82.4)	24.5 (range 6–107)	23.6 (range 6–336)	N/A	17	3.2 (range 1.8–7.3)	2	14	19.5 (range 0–63.9)	N/A (range 6–8)	N/A	N/A	N/A
Zamora et al., 2020 [54]	3	RCCS	14	N/A	N/A	N/A	N/A	1	N/A	2	2	N/A	N/A	N/A	N/A	N/A

Abbreviations: RCT, randomized controlled trial; PCCS, prospective comparative cohort study; RCCS, retrospective comparative cohort study; PCS, prospective case series; RCS, retrospective case series; CoNS, coagulase-negative staphylococci; MSSA/MRSA, methicillin-sensible/resistant *Staphylococcus aureus*; SHC, short-course antibiotic therapy; STD, standard-course antibiotic therapy; PJI, periprosthetic joint infection; FU, follow-up; KSS, Knee Society Score; HSS, Hospital for Special Surgery Knee-Rating Scale; N/A: data not available.

**Table 2:** Data from series reporting on articulated spacers.

Article	Level of Evidence	Study Type	Spacer Details	Patient N° (Spacer N°)	Mean Age (Year)	Mean Follow-Up (Months)	Mean Time to PJI Onset after Implant (Months)	Most Frequent Pathogens	Spacer Exchange: Repeated First Stage	Mean Time between First Stage and Second Stage (Months)	No Reimplantation (*n*°)	PJI Recurrence (*n*°)	Mean Time to Recurrence (Months)	Mean Duration of Antibiotic Therapy (Weeks)	Active Knee Flexion at Last Follow-Up (Degrees)	Functional Outcomes at Last Follow-Up: Score Type and Values	Peri-Operative Non-Infection-Related-Complications (N°)
Ahmad et al., 2013 [55]	3	PCCS	C/C	75	67.5 (range 57–85)	51.6 (range 24–84)	N/A	27.9% *S. aureus*; 25.6% CoNS; 11.6%	0	N/A (range 0.7–5)	1	7	42	N/A (range 4–12)	115 (range 90–125)	KSS: 89.5 (range 74–95)	N/A
Akhtar et al., 2019 [21]	3	RCCS	C/C	13	69	32.9	N/A	N/A	2	9.6	N/A	2	N/A	N/A	N/A	N/A	10
			Pedestal C/C	14	73.4	52.5			1	7.7		1					2
Babis et al., 2008 [56]	4	PCS	C/C	24	71 (range 58–84)	72 (range 24–120)	N/A	58.3% *S. aureus*; 42.9% *S. epidermidis*	0	1.4	0	0	/	6	100	N/A	1
Boelch et al., 2021 [57]	4	RCS	C/C	60	67.8 (range 46–85)	35.6 (range 1–135)	53.5 (range 2–239)	N/A	8	N/A	4	12	N/A	6	N/A	N/A	N/A
Brunnekreef et al., 2013 [23]	3	RCCS	M/P	26	58	12	56.4	N/A	0	4.4	0	0	N/A	6	96.4	N/A	N/A
Buyuk et al., 2017 [58]	4	PCS	C/C	25	70 ± 9.7 (range 52–88)	39.8 ± 12.5 (range 22–73)	N/A	36% MRSE; 12% MSSE	2	3.8 ± 1.4 (range 2–6)	0	1	N/A	9.3 ± 3.4	N/A	KSS: 77 (range 32–96)	3
Carulli et al., 2013 [59]	4	PCS	M/P	9	66.5 (range 59–71)	55.2 (range 48–84)	15.5 (range 5–32)	67% *S. aureus*; 33% *S. epidermidis*	0	1.9 (range 1.6–2.3)	0	0	/	4.2 (range 3–7)	110 (range 105–125)	KSS: 86.4 (range 74–97)	0
Castelli et al., 2014 [60]	4	PCS	C/C	50	68 (range 54–80)	84 (range 24–156)	N/A	46% CoNS; 11% MSSA	0	3.7 (range 0.9–13.8)	0	4	12.8	6	94	KSS: 75.38	0
Chen et al., 2016 [24]	3	RCCS	M/P (autoclaved)	10	68.9 (range 20–88)	32 (range 24–46)	N/A	N/A	0	4.4 (range 2 –9.7)	0	2	13 (range 11–15)	6	94.5 (range 70–125)	KSS: 74.7 (range 62–88)	3
Chiang et al., 2011 [25]	3	PCCS	C/C	23	71 (range 65–78)	N/A	N/A	N/A	0	3.4 (range 2.5–5)	0	1		11.2	113 (range 95–125)	HSS: 90 (range 86–94)	0
DeBoer et al., 2020 [15]	3	RCCS	C/C	77	63 (range 42–83)	N/A (range: 12–120)	N/A	N/A	0	4.4 (range 1.8–18.9)	2	14	N/A	6	N/A	N/A	N/A
Durbhakula et al., 2004 [61]	4	RCS	C/C	24	72 (range 44–94)	33 (range 28–51)	N/A	37.5% *S. epidermidis*; 25% *S. aureus*	0	2.8 (range 2.3–4.4)	2	0	/	6	104 (range 89–122)	HSS: 82 (range 63–96)	2
Evans 2004 [62]	4	RCS	C/C	31	64	>24	N/A	29% MRSA; 25.8% MSSA	0	2.4	2	3	21.3	6	111 (range 0–130)	N/A	1
Fehring et al., 2000 [29]	3	RCCS	C/C	30	N/A	27 (range 24–36)	N/A	N/A	0	N/A	1	1	N/A	6	105 ± 12 (range 90–126)	HSS: 84 ± 13 (range 45–95)	2
Fei et al., 2022 [63]	3	RCCS	C/C	23	67.6 ± 9.4 (range 52–81)	46.6 ± 25.4 (range 14.4–91.3)	N/A	30.4% *S. epidermidis*; 17.4% *S. aureus*	0	3.6 ± 2 (range 2.2–10.9)	4	0	/	6	77.4 ± 9.2 (range 60–90)	KSS: 84.1 ± 5.6 (range 73–93)	0
			M/P (autoclaved)	24	67.8 ± 9.5 (range 37–80)	50.5 ± 28.8 (range 19.1–134.5)		33.3% *S. epidermidis*; 12.5% *S. aureus*	1	6.4 ± 4.6 (range 2.2–20.1)		0	/		85 ± 11.1 (range 60–100)	KSS: 83.4 ± 10 (range 52–93)	1
Freeman et al., 2007 [30]	3	RCCS	C/C	48	64.9	62.2 (range 25.7–119.6)	N/A	N/A	0	N/A	0	4	N/A	6	N/A	KSS: 70 (range 39.5–90)	N/A
Garg et al., 2011 [64]	4	RCS	C/C	36	62 (range 50–76)	62.4	10 (range 7–16)	N/A	0	18 (range 6–42)	7	0	/	N/A (range 10–12)	75.6	N/A	2
Ghanem et al., 2016 [31]	3	RCCS	C/C	30	N/A	N/A	N/A	N/A	N/A	N/A	2	8	N/A	N/A (range 4–6)	N/A	N/A	N/A
Ghanem et al., 2018 [65]	4	RCS	C/C	16	72.0 ± 8.3	22.5 ± 16.6	N/A	37.5% *S. aureus*; 31.2% *S. epidermidis*	0	6.2 ± 5.2	0	4	N/A	N/A (range 4–6)	103.3 ± 17.1	N/A	0
Gooding et al., 2011 [66]	4	RCS	M/P	115	68 (range 35–86)	108 (range 60–144)	N/A	32.2% *S. epidermidis*; 31.3% *S. aureus*	2	3.9 (range 1.2–28.3)	2	14	N/A	> 5	93.2 (range 30–140)	N/A	50
Ha 2006 [67]	4	RCS	C/C	12	65.7 (range 54–73)	N/A (range: 24–42)	N/A	25% MRSA; 16.7% MSSA	0	2.1 (range 0.9–3.7)	0	0	/	N/A	102 (range 75–140)	KSS: 87	8
Haddad et al., 2000 [68]	4	RCS	M/P	45	69 (range 26–83)	48 (range 20–112)	N/A	40% *S. epidermidis*; 20% *S. aureus*	N/A	3.6 (range 0.8–22.3)	1	4	N/A	N/A	94.5 (range 20–135)	HSS: 71.5 (range 32–96)	12
Hammerich et al., 2021 [69]	4	RCS	Reverse C/C (convex tibia + concave femur)	110	67.2 (range 43–89)	N/A	41.0 ± 3.4 (range 1–240)	N/A	3	1.8	0	0	/	N/A	N/A	N/A	0
Hart et al., 2006 [70]	4	RCS	C/C	48	68.2 (range 37.2–81.3)	48.5 (range 26–85)	39.6 (range 5–72)	62.5% CoNS; 10.4% *S. aureus*	0	4.3 (range 1.4–15)	2	6	N/A	2	92 (range 30–120)	N/A	N/A
Hoshino et al., 2021 [71]	4	PCS	C/C	7	77	54 ± 28 (range 11–90)	28 ± 16 (range 10–53)	N/A	0	6 ± 3 (range 3–12)	0	0	/	3	99 ± 22	KSS: 84 ± 10	0
Hsu et al., 2007 [34]	3	RCCS	C/C	21	N/A	58 (range 27–96)	N/A	N/A	0	3.2 (range 1.4–5.5)	0	2	17.3	8.4 (range 6–12)	95 (range 80–120)	KSS: 88.9	N/A
Incavo et al., 2009 [72]	4	RCS	C/C	11	61.1 (range 32–83)	N/A	37 (range 4–108)	45.5% *S. aureus*	0	N/A (range 1.4–5.5)	0	0	/	N/A (range 4–6)	N/A	N/A	2
Jia et al., 2012 [73]	4	RCS	C/C	21	64.4	32.2 (range 17–54)	12.9 (range 8–26)	42.9% *S. epidermidis*; 19% *S. aureus*	1	2.7 (range 1.4–7.4)	0	0	/	4.9 (range 2–8)	94.3	KSS: 82.1	16
Johnson et al., 2012 [38]	3	RCCS	C/C or M/C	34	62 (range 59–65)	27 (range 12–72)	N/A	N/A	N/A	3.1 (range 2.4–3.7)	N/A	6	N/A	N/A	99 (range 60–120)	KSS: 83 (range 48–99)	4
Jung et al., 2022 [74]	3	RCCS	C/C	12	74.5 (range 63–85)	N/A	N/A	25% MSSA; 16.7% *E. coli*	0	1.9 (range 1.4–2.9)	0	0	/	N/A	N/A	N/A	0
			Spiked C/C	15	73.5 (range 60–81)			26.7% MSSA; 20% *E. coli*	1		0	0	/				1
Kalore et al., 2012 [75]	3	RCCS	M/P (autoclaved)	15	67.3	73 (range 37–105)	38.5	37.7% MSSA; 17% MRSA	1	4.9	2	2	N/A	> 6	95.7	N/A	1
			M/P (new)	16	63.6	19 (range 12–32)	31.9		1	2.7	1	1			98.3		0
			C/C	22	61.1	32 (range 14–56)	41.9		0	5.8	0	2			93.8		0
Kong et al., 2021 [39]	3	RCCS	C/C	20	65.5 ± 11.4	18 (range 8–28)	N/A	N/A	1	2.9 (range 2.1–5.1)	0	1	N/A	N/A	94 (range 80–115)	KSS: 75 ± 11.5	4
Kohl et al., 2011 [76]	4	PCS	C/C	16	73.1 (range 54–89)	> 24	N/A	43.8% CoNS; 12.5% *S. aureus*	0	3.5 (range 3–5)	0	0	/	N/A	114 (range 90–125)	KSS: 89.5 (range 78–95)	N/A
Lin et al., 2021 [77]	3	RCCS	C/C	CR: 66	64.4 (range 57–84)	58.3 (range 31–82)	N/A	31.9% *Staphylococcus* spp.; 21.3% *Streptococcus* spp.	5	3.5 (range 2.5–6.4)	4	8	N/A	> 4	N/A	N/A	37
			C/C	PS: 75	67.9 (range 58–87)	56.7 (range 35–81)			10	3.4 (range 2.3–6.0)	3	8					
Lu et al., 2018 [78]	4	RCS	C/P	11	69.9 (range 59–80)	24 (range 12–48)	N/A	63.6% *S. aureus*; 27.2% *S. epidermidis*	0	N/A	0	0	/	6	93.2 (range 80–105)	KSS: 84.9 (range 80–92)	0
MacAvoy et al., 2005 [79]	4	RCS	C/C	13	58 (range 36–71)	28 (range 15–44)	N/A	38.5% *S. epidermidis*; 30.8% *S. aureus*	0	N/A	2	4	/	6	98 (range 45–135)	N/A	5
Macheras et al., 2011 [80]	4	RCS	C/C	34	64 (range 45–73)	145.2 (range 120–168)	N/A	41.1% *S. aureus*; 20.6% *S. epidermidis*	0	N/A	1	3	N/A	6	105 (range 95–120)	KSS: 76 ± 18 (range 58–94)	1
Marothi et al., 2016 [81]	4	RCS	C/C	28	70 (range 56–79)	4	N/A	N/A	0	N/A (range 1.4–1.8)	0	0	/	6	N/A	N/A	2
Mutimer et al., 2009 [82]	4	RCS	C/C	12	71	10	N/A	N/A	0	3.3 (range 2.4–9.0)	0	0	/	6	N/A	N/A	0
Nahhas et al., 2020 [43]	1	RCT	C/C	25	65.7 ± 8.9	42 ± 16.8	N/A	N/A	1	2.5 ± 1.2 (range 2.0–3.0)	1	1	N/A	6	114.0 ± 10.5 (range 109.7–118.3)	KSS: 79.4 ± 17.1 (range 72.4–86.3)	8
Nodzo et al., 2017 [16]	3	RCCS	Preformed C/C	58	65.3 ± 8.6	74.9 ± 35.1	N/A	N/A	N/A	2.5 (range 1.8–3.3)	N/A	10	N/A	6	N/A	N/A	N/A
			Molded C/C	43	66 ± 11.0	43.7 ± 16.7				2.3 (range 1.8–3.2)		5					
			M/P (autoclaved)	39	67.8 ± 10.2	52.4 ± 21.9				2.7 (range 2.2–3.5)		8					
Ocguder et al., 2010 [83]	4	RCS	C/C	17	63 (range 54–75)	20 (range 13–38)	7.7 (range 3–12)	29.4% *Staphylococcus* spp.; 23.5% *S. epidermidis*	0	4.2	1	2	12	6.8 (range 6–10)	85	KSS: 86 (range 40–97)	6
Ortola et al., 2017 [84]	4	RCS	C/C	112	56.2 ± 16.9	32.9 ± 12	36.8 ± 63.6	25.9%, *S. aureus*; 22.3% *S. epidermidis*	7	2.1 ± 0.4	15	3	N/A	N/A	N/A	N/A	N/A
Park et al., 2010 [44]	3	RCCS	C/C	16	60.2 (range 47–72)	29 (range 25–45)	N/A	25% MSSA; 25% C. Albicans	N/A	N/A	N/A	1	N/A	6	108 (range 85–140)	HSS: 87 (range 76–95)	N/A
Pascale et al., 2007 [85]	4	RCS	C/C	14	68 (range 60–76)	N/A	27.6 (range 12–36)	71.4% *S. epidermidis*	0	2.3	0	0	/	9 (range 6–9)	120 (range 97–130)	N/A	0
Pitto et al., 2005 [86]	4	RCS	C/C	21	67 (range 58–89)	24 (range 12–43)	N/A	57.1% *Streptococcu* spp.; 14.2% *S. aureus*	1	3	2	1	N/A	6	94	KSS: 81 (range 30–92)	0
Preobrazhensky et al., 2019 [46]	3	RCCS	M/P (autoclaved)	67	N/A	12	N/A	N/A	1	N/A	0	1	N/A	N/A (range 6–8)	N/A	N/A	N/A
Radoicic et al., 2016 [87]	4	RCS	C/C	18	66.6	N/A	N/A	Multi–bacterial	3	N/A	5	2	N/A	N/A	N/A	N/A	N/A
Roof et al., 2021 [88]	3	RCCS	C/C or M/P (new)	72	63.4 ± 11.7	24	N/A	N/A	6	N/A	5	8	N/A	N/A	93.7 ± 28	N/A	1
Rossi et al., 2021 [47]	3	RCCS	C/C or M/P (autoclaved)	27	N/A	18	N/A	N/A	1	N/A	0	1	N/A	N/A	114.8 ± 28	KSS: 80.8 ±10	1
Sakellariou et al., 2015 [89]	4	PCS	C/C	46	65.3 (range 32–84)	36 (range 8–60)	33.6 (range 4–84)	39.1% *S. aureus*; 26.1% *Streptococcus* spp.	0	N/A	0	6	N/A	N/A	N/A	N/A	3
Schneider et al., 2022 [48]	3	RCCS	M/P (new)	30	65.6 (range 11.4)	N/A	N/A	N/A	N/A	N/A	2	6	N/A	N/A	99.3	N/A	5
			C/C	19	64.6 (range 11.7)	N/A	N/A	N/A	N/A	N/A	2	6	N/A	N/A	77.2	N/A	5
Seo et al., 2020 [90]	4	RCS	C/C	14	70.2 ± 6.3	44.9 ± 6.5	N/A	21.4% *Streptococcus* spp.; 21.4% *S. aureus*	0	N/A	0	0	/	N/A	92.9	N/A	0
Shaikh et al., 2014 [91]	4	RCS	C/C	13	65	48	N/A (range 0.5–18)	15.4% MRSA; 15.4% C. Albicans	1	5.6 (range 2–29)	0	0	/	> 2	115 (range 75–150)	KSS: 83	0
Shen et al., 2010 [92]	4	RCS	C/C	17	67 (range 52–76)	31 (range 18–47)	N/A	23.5% *Streptococcus* spp.; 23.5% *S. aureus*	N/A	7.8	7	1	N/A	> 6	95.4 (range 90–105)	HSS: 83.6	10
Siebel et al., 2002 [93]	4	RCS	C/C	10	66.1	18.1	N/A	20% *S. epidermidis*; 10% *S. aureus*	0	1.9 (range 1.4–2.8)	0	0	/	N/A	86.5	HSS 63.8	0
Skwara et al., 2016 [50]	3	RCCS	C/C	16	N/A	8.5	N/A	N/A	N/A	N/A	N/A	1	N/A	N/A	102 ± 8.4	N/A	0
Struelens et al., 2013 [94]	4	RCS	C/C	154 (155)	66 ± 11	N/A	N/A	N/A	N/A	1.8 ± 0.79	N/A	N/A	N/A	N/A	N/A	N/A	82
Su et al., 2009 [95]	4	RCS	C/C	15	72 (range 65–79)	47.5 (range 37–61)	N/A	60% MRSA; 10% CoNS	0	3	2	1	N/A	N/A	110 (range 95–120)	HSS: 90.5 (range 82–92)	1
Thabe et al., 2007 [96]	4	RCS	C/C	20	72.3 (range 48–83)	73.2	N/A	N/A	0	0.9	0	0	/	N/A	106	N/A	0
Tian et al., 2018 [97]	4	RCS	C/C	25	64.9 (range 56–83)	64.2 (range 52–89)	N/A	20% MRSE; 16% MSSE	0	2.6 (range 1.4–7.3)	0	0	/	N/A	94 (range 90–98)	KSS: 83 (range 80–88)	8
Tigani et al., 2013 [98]	4	PCS	C/C	37 (38)	68 (range 36–86)	65 (range 24–139)	N/A	31.6% MSSE; 15.8% MRSE	5	2.4 (range 1.6–6.9)	2	9	N/A	6	101 (range 80–115)	N/A	1
Tsai et al., 2019 [99]	4	RCS	C/C	32	73.3 (range 58–93)	36.9 (range 30.1–45)	N/A	21.9%, MSSA; 15.6% *Enterococcus* spp.	3	8.8 (range 4–12.5)	1	4	N/A	> 4	102 (range 80–122)	HSS ± 84.2 (range 78–90)	2
Van Thiel et al., 2011 [100]	4	RCS	C/C	60	66 (range 42–91)	35 (range 24–51)	N/A	20% MRSA; 20% MSSA	1	2.7	1	7	16.3 (range 2–30)	N/A	101.3 ± 18	KSS: 78.6 ± 17.8	1
Vasarhelyi et al., 2022 [52]	3	RCCS	C/C	104	68.6 ± 10.6	120 ± 49.2	43.8 (range 3–168)	N/A	7	3	N/A	17	N/A	6	110.6 ± 13.5	KSS: 86.8 ± 13.6	4
Vasso et al., 2016 [101]	4	RCS	C/C	46	69 (range 58–84)	144 (range 72–192)	N/A	37% MSSA; 28.3% CoNS	2	2.5 (range 2.3–3.1)	N/A	0	/	8	115 (range 100–128)	N/A	0
Vecchini et al., 2017 [102]	4	PCS	C/C	19 (20)	65.4 (range 30–82)	74.1 (range 10–112)	N/A	60% MSSA; 20% MRSA	0	9.1 (range 3–27)	1	0	/	3.6 (range 2–5)	N/A	N/A	4
Villanueva-Martinez et al., 2008 [103]	4	RCS	C/C	30	71 (range 64–82)	36 (range 24–60)	18 (range 1–144)	40% CoNS; 30% MSSA	1	3.5	1	N/A	N/A	N/A	107 (range 90–120)	N/A	6
Wan et al., 2012 [104]	4	RCS	C/C	33	70 ± 11	44 (range 24–62)	41 (range 1–192)	24.2% MSSA; 24.2% CoNS	8	3.2 (range 1.84–7.31)	2	3	N/A	6	N/A	N/A	N/A
Yi et al., 2015 [105]	4	RCS	C/C	17	63.7 (range 43–74)	45.6 (range 24–96)	N/A	23.5% *S. epidermidis*; 11.8% MSSA	1	3.9 (range 2.3–6.2)	1	1	N/A	4	105.9 (range 90–125)	HSS: 83.9 (range 77–91)	N/A
Zamora et al., 2020 [54]	3	RCCS	M/P (new)	3	N/A	N/A	N/A	N/A	1	N/A	N/A	0	/	N/A	N/A	N/A	N/A

Abbreviations: RCT, randomized controlled trial; PCCS, prospective comparative cohort study; RCCS, retrospective comparative cohort study; PCS, prospective case series; RCS, retrospective case series; C/C, cement on cement; C/P, cement on polyethylene; M/P, metal on polyethylene; M/C, metal on cement; CoNS, coagulase-negative staphylococci; MSSA/MRSA, methicillin-sensible/resistant *Staphylococcus aureus*; MSSE/MRSE, methicillin-sensible/resistant *Streptococcus* *epidermidis*; PJI, periprosthetic joint infection; CR, cruciate-retaining total knee arthroplasty; PS, posterior-stabilized total knee arthroplasty; FU, follow-up; KSS, Knee Society Score; HSS, Hospital for Special Surgery Knee-Rating Scale; N/A: data not available.

**Table 3:** Summarized data from the included studies of this review.

	Static	Spacers with Data Available (*n*)	Articulated	Spacers with Data Available (*n*)	*p*-Value
Study series (*n*) RCT PCCS RCCS PCS RCS	34 1 2 19 0 12	-	71 1 2 23 9 36	-	0.111
Spacers (*n*)	1511	-	2739	-	-
Mean age (years)	67 ± 5.6	1147	66.4 ± 3.5	2545	0.532
Mean follow-up (months)	68 ± 52.3	1002	53.5 ± 32.9	2163	0.117
Most frequent bacterial population	*S. aureus* CoNS *S. epidermidis*	347	*S. aureus* *S. epidermidis* CoNS	1303	-
Mean time to PJI (months)	34.8 ± 14.3	296	36.8 ± 11.9	737	0.735
Mean time between first and second stage (months)	3.1 ± 1.1	854	3.6 ± 2.3	2071	0.480
Mean duration of antibiotic therapy (weeks)	7.2 ± 1.9	870	6.1 ± 1	1170	0.007
Repeated first stage/spacer exchange (*n*)	47 (5.4%)	922	89 (4%)	2237	0.159
No reimplantation (*n*)	67 (7.1%)	947	94 (4.3%)	2198	0.001 *
PJI recurrence (*n*)	157 (12.4%)	1271	230 (9%)	2554	0.001 *
Mean time to PJI recurrence (months)	13.7 ± 3.9	285	23.2 ± 12.1	737	0.125
Mean active knee flexion at last FU	91.6 ± 7	763	100.3 ± 9.9	1549	<0.001 *
Mean KSS score at last FU	81.1 ± 13.1	569	81.9 ± 5.5	732	0.792
Mean HSS score at last FU	81.8 ± 0.7	67	81.7 ± 7	229	0.981
Peri-operative non-infection-related local complications (*nn*)	146 (16.7%)	872	318 (16.5%)	1932	0.852
Non-infection-related complications requiring revision surgery (*n*)	24 (2.9%)	820	58 (3.1%)	1876	0.819

* Statistically significant. Abbreviations: RCT, randomized controlled trial; PCCS, prospective comparative cohort study; RCCS, retrospective comparative cohort study; PCS, prospective case series; RCS, retrospective case series; CoNS, coagulase-negative staphylococci; PJI, periprosthetic joint infection; FU, follow-up; KSS, Knee Society Score; HSS, Hospital for Special Surgery Knee-Rating Scale.

**Table 4:** Correlations between time to second stage and outcomes.

	Static		Articulated		Total		Spacers with Data Available (*n*)
	Rho	*p*-Value	Rho	*p*-Value	Rho	*p*-Value	
PJI recurrence (*n*)	0.040	0.876	0.274	0.040 *	0.202	0.082	2786
Mean time to PJI recurrence	0.5	0.391	−0.772	0.072	0.092	0.789	474
Mean active knee flexion at last FU	−0.080	0.595	−0.361	0.019 *	−0.257	0.050 *	1656
Mean KSS score at last FU	−0.267	0.455	−0.073	0.759	−0.147	0.438	956

* Statistically significant. Abbreviations: PJI, periprosthetic joint infection; KSS, Knee Society Score; FU, follow-up.

## Data Availability

The data reported in this study are available in the literature.
